# Methane emissions from upstream oil and gas production in Canada are underestimated

**DOI:** 10.1038/s41598-021-87610-3

**Published:** 2021-04-13

**Authors:** Katlyn MacKay, Martin Lavoie, Evelise Bourlon, Emmaline Atherton, Elizabeth O’Connell, Jennifer Baillie, Chelsea Fougère, David Risk

**Affiliations:** 1 Department of Earth Sciences, St. Francis Xavier University, Antigonish, NS Canada; 2 Department of Engineering and Applied Science, Memorial University of Newfoundland, St. John’s, Newfoundland Canada

**Keywords:** Environmental monitoring, Environmental impact, Climate-change policy

## Abstract

Methane emissions were measured at 6650 sites across six major oil and gas producing regions in Canada to examine regional emission trends, and to derive an inventory estimate for Canada’s upstream oil and gas sector. Emissions varied by fluid type and geographic region, with the heavy oil region of Lloydminster ranking highest on both absolute and intensity-based scales. Emission intensities varied widely for natural gas production, where older, low-producing developments such as Medicine Hat, Alberta showed high emission intensities, and newer developments in Montney, British Columbia showed emission intensities that are amongst the lowest in North America*.* Overall, we estimate that the Canadian upstream oil and gas methane inventory is underestimated by a factor of 1.5, which is consistent with previous studies of individual regions.

## Introduction

Reducing methane (CH_4_) emissions from anthropogenic activities is a critical part of climate change mitigation efforts^1^. Although atmospheric CH_4_ concentrations are low (~ 1.8 ppm)^2^, the warming potential of CH_4_ is 84 times higher than that of carbon dioxide over a 20-year timeframe^3^, making it an immediate target for greenhouse gas (GHG) reductions.

Canada’s second most abundant greenhouse gas is CH_4_, making up 13% of national GHG emissions^4^. In 2018, 43% of Canada’s anthropogenic CH_4_ emissions originated from oil and gas systems^4^. The major sources of oil and gas CH_4_ emissions are from activities that occur during upstream production, which include venting (intentional releases; ~ 52%), incomplete combustion during flaring (~ 1.4%), and fugitive emissions (unintentional releases from faulty equipment, or drilling; ~ 42%)^4^. In response to the climate crisis, Canada’s federal government committed to reducing CH_4_ emissions from the oil and gas sector 40–45% below 2012 levels by 2025^5^. Although the federal government drafted regulations to achieve these reductions^5^, provincial governments in Alberta, Saskatchewan, and British Columbia have also proposed their own regulations to achieve equivalent reduction goals, which have since received approval to replace the original federal regulations^6–8^.

Canada’s CH_4_ reduction targets are based on component-level inventory estimates reported annually in the national inventory report (NIR), which are based in part on industry self-estimation and self-reporting^4^. Field measurement studies in Canada and the US have shown that actual emissions range from equivalent to substantially higher than inventory estimates^9–16^. But a national understanding of discrepancies is lacking because most measurement studies in Canada consist of relatively region-specific sample populations which may not be extensible to regions with varying extractive techniques, geology, and geochemical properties. Different emission measurement techniques and technologies, applied at varying scales, also make comparisons difficult.

How do upstream CH_4_ emissions and intensities vary across major oil and gas producing regions in Canada, and how do they compare to the federal inventory? We addressed this question by aggregating site-level emission data collected during nine extensive vehicle-based measurement campaigns across six prominent oil and gas regions in Canada: Montney (British Columbia), Medicine Hat (Alberta), Lloydminster (Alberta & Saskatchewan), Peace River (Alberta), Red Deer (Alberta), and southeastern Saskatchewan. Measurements were collected between 2015 and 2018, with some regions (Lloydminster, Peace River) visited on more than one occasion. These six regions (Fig. S1) include ~ 20% of the non-oilsands producing sites in western Canada. Results from four of these campaigns have already been published^17,18^, but this is the first time the 6650 emission rate estimates have been aggregated. This study represents the most regionally nuanced estimate of upstream Canadian oil and gas fugitive and vented CH_4_ emissions to date, and uses a much larger sample population than the ~ 300 site survey studies used by the Canadian industry to calibrate its upstream CH_4_ inventory^19^.

### Emissions vary by fluid type (oil vs. gas), and geographic region

**Figure 1:**
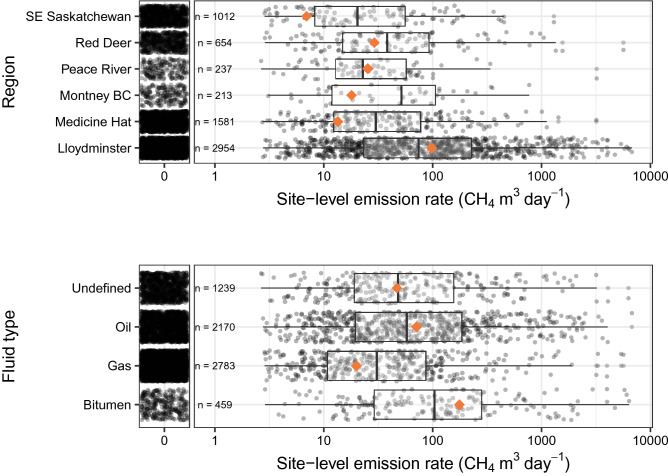
Distributions of measured emission rates (logarithmic scale) by region (top) and by fluid type (bottom). Black dots represent individual site-level emission rates. For better visualization of the emission rate distributions, the plots are broken down to show non-emitting sites (left panel, emission rate = 0), and emitting sites (right panels). The box limits are the interquartile ranges (IQR), and whiskers represent the upper (Q3 + 1.5 * IQR) and lower (Q1 − 1.5 * IQR) ranges of non-zero emissions. The orange diamond is the overall mean (emitting and non-emitting) for each subpopulation.

Site-level measurements show that emissions vary by fluid type and geographic region (Fig. 1). This variability has been documented in recent Canadian studies, at both regional^10,17^, and component-level scales^12,19,20^, as a function of several determinants. In no particular order, the first determinant is the fluid type. Extraction techniques and infrastructure can vary depending on the hydrocarbon produced, which affects emission levels. Sites producing gas had lower average emission rates compared to oil-producing sites, and the overall average emission rate for oil sites was roughly 3.6 times higher than the overall average for gas sites (71.1 m^3^/day/site vs. 19.9 m^3^/day/site) (Fig. 1).

In many oil-producing regions, CH_4_ gas is routinely considered a byproduct and vented because the economics of conservation are unfavorable^21^*.* Additionally, some in-situ heavy oil production processes such as Cold Heavy Oil Production with Sand (CHOPS) generally yield higher rates of routine venting^10–12,17,22^; this is evident in Lloydminster, where CHOPS is the dominant production technique (Fig. 1).

Regulation is another factor that influences regional variability in CH_4_ emission rates. Some geographies are subject to more stringent regulations due to historical air quality violations or other health and safety concerns. For instance, special regulations were enacted in 2017 for the Peace River area because of historical air quality issues, and in recent years producers in the area have reportedly eliminated all venting^17,23^. Our measurements showed average site-level emission rates in Peace River decreased nearly three-fold from 2016 (31.5 m^3^/day/site) to 2018 (11.1 m^3^/day/site) (Table S4), which suggests that these new regulations are resulting in significant mitigation success in this area. Sour (H_2_S-bearing) fields are another example of regulatory success; since H_2_S is a serious health risk, sour developments like SE Saskatchewan have more restrictions on venting, which inadvertently aids in CH_4_ mitigation since the gases are co-emitted^18,24^. SE Saskatchewan had the lowest average site-level emission rate out of all regions included in this study (Fig. 1, Table S4). Effective mitigation depends on an understanding of these determinants.

### Current component-level inventory is underestimated

Various authors have pointed out systematic biases in the component-level inventory process (as used in federal reporting), especially the propensity to miss rare large emitters^16,25,26^. To estimate the degree to which the current Canadian upstream CH_4_ inventory is underestimated, we calculated site-level Emission Factors (EFs) from our measurements and applied them to all non-oilsands producing sites in Alberta. Site-level EFs are different than component-level EFs in that they represent an average of aggregate emissions for an oil/gas site (multiple pieces of infrastructure), whereas component-level EFs are average emissions for specific leaking components (e.g. valves, hatches).

To capture the variability in emissions across sites and regions, site-level EFs were calculated for every unique combination of site type and region (Methods Sect. 2.1). Then, we used a Monte Carlo analysis to estimate a total inventory for Alberta (Methods Sect. 2.2). Alberta was chosen for this exercise because the vast majority of our measurements were collected in this province, and because it represents 80% and 67% of total Canadian oil and gas production, respectively^27^.

Our measurement-based inventory indicates that the non-oilsands upstream oil and gas sector in Alberta emitted 5,074,449 m^3^ CH_4_/day in 2018 (2.5 percentile = 3,741,309 m^3^/day; 97.5 percentile = 7,453,798 m^3^/day), which is about 1.5 times the most recent component-level inventory of 3,408,534 m^3^ CH_4_/day, derived by Environment and Climate Change Canada for Alberta in 2018^4^. Our findings are consistent with previous CH_4_ emission studies within Canadian developments; no studies have yet identified a Canadian oil and gas producing region with emissions lower than the inventory estimate. In previous studies, factors of 1–15 have been estimated, with most being in the range of 1.5–3.0^9–11,14,15^. This implies that CH_4_ abatement costs could be lower per ton of CO_2_ equivalent than previously reported, due to higher volumes of CH_4_ (i.e. profitable natural gas) present at oil and gas sites^28^.

### Emission intensities vary substantially

**Figure 2:**
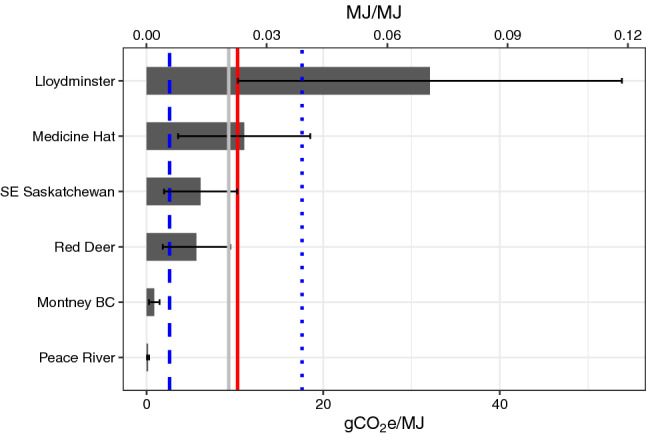
Emission intensities for each region included in this study (horizontal grey bars). The top axis shows intensities as a function of megajoule emitted per megajoule produced (MJ/MJ), and the bottom axis displays intensities as a function of grams of CO_2_ equivalent emitted per megajoule produced (gCO_2_e/MJ). Emission intensity uncertainty ranges are represented via the black error bars, which includes uncertainty from both emission rate and production values. The solid grey vertical line is the overall average from this study, the solid vertical red line is the global lifecycle (well-to-refinery, including all greenhouse gases) average reported in Masnadi et al. (2018)^30^, the dotted blue line shows their estimate for Canada (based on data from 84 oil fields), and the blue thick dashed line is their global average intensity for CH_4_ only^30^.

Emissions intensities for each region were calculated based on measured emission rates and reported production volumes for sampled infrastructure (Methods Sect. 3.1). Emission intensities are expressed using two ratios: (1) Average megajoule emitted per megajoule produced (MJ/MJ) (Fig. 2); and (2) grams of CO_2_ equivalent emitted per megajoule produced (gCO_2_e/MJ) (Fig. 2). All emission and production values used in this analysis can be found in Table S4. The average production volumes used in this calculation were from the same month in which the measurements were acquired. For this reason, intensities were calculated individually for each campaign, and then averaged for regions sampled on more than one occasion (Lloydminster and Peace River). Also, it is important to note that our emissions intensity calculations do not include all life-cycle (“well-to-wheel”) emissions from these hydrocarbon sources, but focuses on those emitting directly during upstream production (i.e. scope 1 emissions).

Average emissions intensities across regions vary significantly, ranging from 0.0004 ± 0.0003 (Peace River) to 0.0706 ± 0.0479 (Lloydminster) (Fig. 2, Table S3). In Fig. 2 we can see that Lloydminster heavy oil ranks highest in intensity, with roughly 7% of the energy produced being lost via fugitives and vents. This finding was somewhat expected, given the high average emission rates in this region (Table S4). Interestingly, however, our analysis also found that Medicine Hat ranked second highest in intensity (0.0243 ± 0.0165), despite having a relatively low average emission rate per site (Table S4). We see high intensities in Medicine Hat because production rates in this region are so low (Table S4). Although Medicine Hat has the highest density of wells in Alberta (Table S2), the region only accounts for a small portion (~ 7%) of the province’s gas production^12^. Such findings are important, because aggregate production, transmission, and distribution leaks here (and potentially in other old and low-producing developments) could conceivably approach overall leak rates of 3.2%—where natural gas is estimated to approach the climate warming impact of coal^29^. If so, these developments could become increasingly exposed to market or investment barriers, as investors and consumers move towards fuels with less embodied carbon.

Our estimated emissions intensities (in gCO_2_e/MJ) for each region can be readily compared with those recently published in Masnadi et al. (2018)^30^. In that study, authors calculated full life-cycle (well-to-refinery) emissions intensities for hundreds of oilfields around the world^30^. Using the best available data and the OPGEE model, Masnadi et al. (2018)^30^ found a global carbon intensity life-cycle average of 10.3 gCO_2_e/MJ (+ 6.7, − 1.7, 95% CI), of which 2.6 gCO_2_e/MJ was exclusively derived from CH_4_ emissions. For 84 Canadian oilfields in the study, the overall average carbon intensity was 17.6 gCO_2_e/MJ^30^. With the exception of Lloydminster, all of our intensity estimates are lower than their average for Canadian oilfields (Fig. 2), which was expected since our estimates only consider the CH_4_ emissions component of total life-cycle carbon. However, our Lloydminster and Medicine Hat CH_4_-only intensities exceeded Masnadi et al. (2018)’s global average for total carbon life-cycle emissions^30^ (Fig. 2). In these regions, actual full life-cycle emissions may significantly outstrip the global average. On the other hand, we also found that the Montney BC and Peace River regions have extremely low CH_4_ emission intensities that fall well below the global and Canadian averages, suggesting that these regions produce oil and gas more efficiently with respect to CH_4_ leakage. Additionally, emission intensities for all producing regions in Canada, except for Lloydminster and Medicine Hat, were lower than the US average of 2.3% (of gross production) recently reported by Alvarez et al. (2018)^16^.

In conclusion, there is significant variability in absolute CH_4_ emissions and emission intensities across major oil and gas regions in Canada. As seen in previous studies, Lloydminster is an area characteristic of high CH_4_ emissions. Fortunately, new regulations should address some of these prominent emission sources (especially vented emissions), and future work in this area could verify regulation-driven reductions. Our emissions intensity analysis revealed that low producing regions like Medicine Hat have high intensities, which has both environmental and economic implications that should be considered as we move towards a low-carbon future. In contrast, Montney BC and Peace River regions showed extremely low emission intensities, making natural gas produced here an attractive investment for companies with Environment, Social, and Governance (ESG) standards. Lastly, CH_4_ emissions from the oil and gas sector in Canada likely exceed inventories by a factor of 1.5. Because conserved CH_4_ is saleable, this implies that reduction costs per ton could be less than previously estimated^28^. Increased measurement and reporting requirements as a result of new regulations should be used to inform future inventory estimates, to ensure annual reductions are accurately estimated.

## Materials and methods

### Data acquisition and processing

#### Overview

Our methods are broken down into a four-step process involving data acquisition, plume detection, attribution, and emission rate estimation: (1) Data was collected via extensive truck-based surveys of air composition measuring three or more atmospheric gases at ppb-levels, geo-located, at 1 Hz frequency. Gas concentrations (CH_4_, CO_2_, C_2_H_6_, H_2_S) were measured in real time using laser spectrometers (Picarro Inc., Santa Clara, CA, USA), (2) computational signal processing and geochemical analysis were used to distinguish oil and gas emissions from biogenic, naturally occurring sources, or other anthropogenic emissions, (3) we conducted a back-trajectory analysis to attribute emission anomalies observed on-road to specific upwind sites, (4) volumetric emissions rates were estimated via a point-source Gaussian Dispersion Model (GDM). Each of these steps, and uncertainties therein, have been explained previously^9,17,18^. Thus, only a brief overview is included in the next three subsections.

Table S1 provides basic statistics (dates, number of surveys, distance) for all measurement campaigns included in this analysis. Although some of these individual campaigns were the focus of previous peer-reviewed studies, all measurements were conducted by our research group using the same equipment and survey protocols, which allowed for uniform processing and analysis of the data for this work. It should also be noted that measurements include active and suspended sites only, as emissions from abandoned infrastructure were not the focus of these studies. Short-lived emissions from intermittent activities like exploration and drilling are also not included (i.e. measured emissions represent emissions during production only). Measurements were taken in six contrasting regions across the three major oil and gas producing provinces in Canada (British Columbia, Alberta, and Saskatchewan). Figure S1 is a map with polygons depicting the geographical boundaries covered in this study. A total of nine vehicle-based measurement campaigns were completed (with some regions being surveyed more than once). All campaigns were conducted on public roads without giving notice to any operators or regulators in the regions. Preplanned survey routes were driven multiple times (often on different days) and were designed to target areas with dense infrastructure. Table S2 describes general profiles for each region, including the type of hydrocarbon produced, primary production styles, and approximate active well counts as of January 2020.

### Geochemical and geospatial analysis

To identify thermogenic methane (CH_4_) plumes, we analyzed ratios of super-ambient CO_2_ and CH_4_ concentrations, as opposed to raw atmospheric concentrations which are more prone to false characterization. To do this, we first used an adaptive algorithm to establish background concentrations of each gas, which accounts for the spatiotemporal variability observed on multi-hour surveys. From there, we subtracted these background concentrations to calculate excess ratios (hereafter referred to as e-ratios). These e-ratios act as a geochemical fingerprint and were used to identify areas of CH_4_-enrichment. They were also used to distinguish between different emission sources (e.g. from natural sources or engine combustion). For this study, we used an eCO_2_:eCH_4_ threshold of < 100 to indicate thermogenic CH_4_ plumes. Such measurements of CH_4_-enrichment needed to persist for more than three consecutive (1 Hz) measurements to be considered a thermogenic plume (i.e. if there was one measurement that fell below the e-ratio threshold, but the following measurement was above the threshold, then the first measurement was not considered to be from a plume). During surveys, time-series measurements were collected every second.

Once the plumes were geospatially located, we used back trajectory analysis to attribute the plumes observed on road to upstream infrastructure sites. Here, an infrastructure site is defined as all pieces of infrastructure at upstream oil and gas production sites (wells and facilities), that exist within a 45 m radius of each other. Sites were considered sampled when at least two sequences of measurements (i.e. “passes”) were taken < 500 m downwind (i.e. it was passed downwind at least twice). Sites were considered to be emitting only if a CH_4_ plume was detected < 500 m downwind on more than 50% of passes. If multiple sites fell within 500 m of a plume, the closest site was tagged as the emission source.

### Volumetric CH_4_ emission rate estimates using inverse Gaussian dispersion model

After geochemical and geospatial attribution, we estimated emission rates for all sites tagged as emitting. To do this, we used a point-source Gaussian Dispersion model, which incorporates both measured and estimated parameters including downwind CH_4_ concentration, wind speed, measurement-to-source distance, emission source height, and Pasquill atmospheric stability. Since most sites consist of multiple pieces of infrastructure, and this methodology cannot confidently attribute plumes to a single well or facility, we estimated emission rates for all individual infrastructure within each site, which considers variable equipment (i.e. potential leak source) heights. We then used the median emission rate per site for all subsequent analyses. Reasons for using the median rather than the mean are discussed further in the next section.

### Measurement uncertainty

The uncertainties related to our methods of plume detection, attribution, and emission rate estimation have been previously evaluated^9,17,18^. Plume detection uncertainty (i.e. the probability of detecting false positives) was estimated to be < 1%, whereas attribution uncertainty was estimated to range from 7.5 to 33% (depending on infrastructure density). Emission rate estimates represent our largest source of uncertainty, which are described extensively in O’Connell et al. (2019)’s Supplemental Material^17^. O’Connell et al. (2019) documented an emission rate estimate uncertainty (standard error) of ± 63%, which was calculated using controlled release experiments conducted over five days, under a range of atmospheric conditions^17^. Results from these experiments also revealed an upward bias of 30% for mean emission rates measured by three passes, but the median value was found to be less skewed^17^. For these reasons (as noted above), the median emission rate for each site was used in this analysis, to ensure a more conservative, unbiased estimate. These emission rate uncertainties are comparable to those documented in other transect-based Gaussian dispersion model studies^31,32^.

### Fluid type classification

Fluid types for all measured sites were classified as “Oil”, “Bitumen”, “Gas”, or “Undefined” based on their infrastructure description (Fig. 1). For example, a “Crude oil single well battery” site was classified as an oil site. If oil, bitumen, or gas were not included in the site description, then the site was classified as “Undefined”. Out of all 6651 measured sites, 1239 were classified as “undefined.”

## Site-level emission factor calculations and Alberta CH_4_ inventory estimate

### Emission factor calculations

We calculated site-level Emission Factors (EFs) using our measurements and applied them to all non-oilsands producing sites in Alberta to derive an overall CH_4_ inventory estimate. Oilsands sites were excluded as we lacked measurements for these facilities (these sites are not ideal for vehicle-based measurement techniques). EFs were derived by calculating the mean emission rate for all unique combinations of infrastructure types and regions, which we define as Type-Region (TR) bins. For example, Single wells in Medicine Hat would represent a unique TR bin. All emission rate measurements for active sites (including those measured as 0) were included in the calculations. We used the ten Alberta Energy Regulator (AER) administrative regions (Fig. S2) as the physical boundaries in which measurements were considered for region-specific EFs (excluding the oilsands dominant Fort McMurray region). Using the previous example, an EF for TR bin Single well-Medicine Hat is the average of all emission rates (including sites measured as non-emitting, i.e. emission rate = 0) for single well sites within the Medicine Hat region (Fig. S2). It is important to note that while using this method, a type of infrastructure site could have multiple EFs across different regions. For example, an EF for a single well in Medicine Hat might be different than an EF for a single well in Red Deer (since they would each represent a unique TR bin). If a certain infrastructure site type was not sampled in a particular region, an EF was derived by averaging all measurements (from all regions) of that site type.

We chose to calculate EFs separately for all unique TR bins because we know from previous studies^10–12,14,17,24^ that emissions can vary significantly based on these two factors. Our method lets us account for the variability that exists within the upstream sector, which in turn helps avoid scenarios of over and underestimations. A full list of EFs used in the total inventory estimate is in Table S5.

### Alberta CH_4_ inventory estimate and uncertainty

To estimate an overall methane inventory for Alberta upstream oil and gas production, we first needed to calculate the total number of oil and gas sites in the province (excluding oilsands). IHS databases (IHS Markit) (Table S5) were used to determine site counts. Since infrastructure data in IHS databases are not aggregated to site-level, we grouped individual wells and facilities that fell within a 45 m radius of one another to determine total site counts. This step is required because our EFs correspond to a site-level estimate. Then, we subset our infrastructure dataset to only include sites that were either producing, venting, or flaring hydrocarbons during the 2018 production year (according to publicly available Petrinex reporting data^33^). Finally, this dataset was used to calculate individual site counts for each TR bin (Table S5).

From there, we used a Monte Carlo analysis to estimate the total Alberta inventory and 95% CI. For each TR bin, we created a probability density function (pdf) with a lognormal distribution (mean = EF, n = 10,000, SD =  ± 63%). A lognormal fit was chosen as previous studies have shown emissions to follow this distribution^13,14,16–18,25^. Then, a random value from each pdf is sampled, and multiplied by the corresponding TR bin site count, resulting in a total emission estimate for each TR bin. Totals from all TR bins are then summed to compute a total provincial inventory. This process was repeated 10,000 times across all TR bins, resulting in a distribution of total inventory estimates (n = 10,000), with a mean value of 5,074,449 m^3^ CH_4_/day, and 95% of values falling between 3,741,309 and 7,453,798 m^3^/day. Using this method, we were able to incorporate the “heavy-tail” of the emissions distribution, as well as our measurement uncertainty into the total estimate. We assumed infrastructure count uncertainty to be negligible.

## Emissions intensity analysis

### Calculations

Since there are no standard units to calculate emission intensities, we expressed our estimates using two ratios: (1) Average megajoule emitted per megajoule produced (MJ/MJ), and (2) grams of CO_2_ equivalent emitted per megajoule produced (gCO_2_e/MJ).

To calculate the amount of energy (MJ) emitted for each region, we first calculated the cumulative CH_4_ emission rate (in m^3^/day) for each campaign (i.e. summed all site-level emissions that were measured over each campaign). Cumulative emission rates for each campaign are shown in Table S4. These cumulative emissions were converted to megajoules (MJ) using a conversion of 1 m^3^ CH_4_ = 37.3 MJ, which is based on 1000 Btu/cf^34^. We converted emissions (in m^3^/day) to grams of CO_2_e using a global warming potential (GWP) of 25 (over 100 years), and a density of 678 g/m^3^ (15 °C, 1 atm) for CH_4_.

To calculate the average energy produced per day at all measured sites, we extracted aggregated production data from IHS databases. Complete lists of all sampled wells during each campaign were imported to IHS Markit software (AccuMap) to get specific production data for the same sites that were measured for emissions. Daily average production rates for all producing wells in the sampled well lists were extracted and then summed to get a combined average production rate per day per region. In other words, average daily production rates for all sampled wells were summed to get a combined daily average production rate. This was done separately for both oil (m^3^/day) and gas (10^3^m^3^/day), and production data used in these calculations corresponded to the same month(s) in which the sites were measured for emissions. Consequently, our production rates are based on a small subset of wells relative to total infrastructure counts in these areas (especially when many of the sampled wells were not producing), and as a result, these production values may not be representative of the entire regions. However, we do believe this method was the best way to ensure we were getting site- and time-specific production values for actual wells that were measured for emissions.

From there, the combined daily average production rates for oil and gas were converted to megajoules (MJ). For oil production, we used a conversion of 1 m^3^ = 38,510 MJ for light oil, and 1 m^3^ = 40,900 MJ for heavy oil^34^. For gas production, we used the same conversion rate used to convert CH_4_ emissions to energy units (MJ) (see above). These values were then summed to get a single value representing the average energy produced per day for all sampled sites.

Finally, the daily energy (MJ) emitted and daily gCO_2_e emitted values were divided by the average daily energy produced (MJ), for all sites sampled for each campaign, resulting in a single emission intensity value for each measurement campaign. For regions sampled across multiple campaigns (Lloydminster and Peace River), final intensities were averaged to get a single value per region.

### Emission intensity uncertainty

We quantified uncertainties in our intensity calculations, which considered uncertainties for both emission rate estimates and production volumes. Average emission rate uncertainties were estimated to be ± 63%. This uncertainty was discussed earlier in Sect. 1.4 and is explained in O’Connell et al. (2019)^17^. For production volume uncertainties, we assumed an average production volume uncertainty of ± 25%, which was based on values published in Table 13 of Clearstone Engineering’s inventory methodology report (same methodology used for Canada’s national inventory reporting estimates)^35^. The overall emissions intensity uncertainty was calculated by combining the uncertainty in emission rates and production volumes using the following error propagation equation:

$$Utotal = \sqrt{{U}_{1}^{2}+{U}_{2}^{2}\dots +{U}_{n}^{2}}$$

where U_1_, U_2_ are the percent uncertainties for each value (emission rates and production volumes). The U_total_ value was then used to determine the upper and lower bounds for each emission intensity estimate (Table S3).

## Supplementary Information

Supplementary Information.

## Data Availability

All emissions data included in this analysis is available (in csv format) for public download.

## References

[1] Rogelj, J. *et al.* Mitigation pathways compatible with 1.5°C in the context of Sustainable Development. https://www.ipcc.ch/sr15/chapter/chapter-2/ (2018)

[2] Dlugokencky, E. Trends in Atmospheric Methane (NOAA/ESRL), esrl.noaa.gov/gmd/ccgg/trends_ch4/ (2020).

[3] Myhre, G. *et al*. Anthropogenic and Natural Radiative Forcing, (Chapter in Climate Change 2013: The Physical Science Basis. Contribution of Working Group I to the Fifth Assessment Report of the Intergovernmental Panel on Climate Change http://www.climatechange2013.org/images/report/WG1AR5_Chapter08_FINAL.pdf (2013).

[4] Environment and Climate Change Canada (ECCC). National Inventory Report 1990–2018: Greenhouse Gas Sources and Sinks in Canada (2020).

[5] Environment and Climate Change Canada (ECCC). Technical backgrounder: Federal methane regulations for the upstream oil and gas sector, https://www.canada.ca/en/environment-climate-change/news/2018/04/federal-methane-regulations-for-the-upstream-oil-and-gas-sector.html (2018).

[6] Alberta Energy Regulator (AER). Directive 060, https://www.aer.ca/documents/directives/Directive060_2020.pdf (2020).

[7] Saskatchewan Ministry of Economy. The Oil and Gas Emissions Management Regulations: Chapter O-2 Reg 7, https://www.canlii.org/en/sk/laws/regu/rrs-c-o-2-reg-7/latest/rrs-c-o-2-reg-7.html (2019).

[8] Province of British Columbia. Oil and Gas Activities Act, http://www.bclaws.ca/civix/document/id/regulationbulletin/regulationbulletin/Reg286_2018 (2018).

[9] Atherton E (2017). Mobile measurement of methane emissions from natural gas developments in northeastern British Columbia, Canada. Atmos. Chem. Phys..

[10] Johnson M, Tyner D, Conley S, Schwietzke S, Zavala-Araiza D (2017). Comparisons of airborne measurements and inventory estimates of methane emissions in the Alberta upstream oil and gas sector. Environ. Sci. Technol..

[11] Roscioli JR (2018). Characterization of methane emissions from five cold heavy oil production with sands (CHOPS) facilities. J. Air Waste Manag. Assoc..

[12] Greenpath Energy. Alberta Fugitive and Vented Emissions Inventory Study, https://www.aer.ca/documents/GreenPathAER%20Survey-Methane.pdf (2016).

[13] Zavala-Araiza D (2015). Reconciling divergent estimates of oil and gas methane emissions. Proc. Natl. Acad. Sci. USA.

[14] Zavala-Araiza D (2018). Methane emissions from oil and gas production sites in Alberta, Canada. Elem. Sci. Anth..

[15] Baray S (2018). Quantification of methane sources in the Athabasca Oil Sands Region of Alberta by aircraft mass balance. Atmos. Chem. Phys..

[16] Alvarez RA (2018). Assessment of methane emissions from the U.S. oil and gas supply chain. Science.

[17] O’Connell E (2019). Methane emissions from contrasting production regions within Alberta, Canada: implications under incoming federal methane regulations. Elem. Sci. Anth..

[18] Baillie J (2019). Methane emissions from conventional and unconventional oil and gas production sites in southeastern Saskatchewan, Canada. Environ. Res. Commun..

[19] Clearstone Engineering Ltd. Update of equipment, component and fugitive emission factors for Alberta upstream oil and gas https://www.aer.ca/documents/UpdateofEquipmentComponentandFugitiveEmissionFactorsforAlber-1.pdf. (2018).

[20] Ravikumar AP (2020). Repeated leak detection and repair surveys reduce methane emissions over a scale of years. Environ. Res. Lett..

[21] Johnson MR, Coderre AR (2012). Opportunities for CO_2_ equivalent emissions reductions via flare and vent mitigation: a case study for Alberta, Canada. Int. J. Greenh. Gas Control.

[22] Alberta Energy Regulator (AER) Upstream petroleum industry flaring and venting report: Industry performance for year ending December 31, 2018, https://www.aer.ca/documents/sts/ST60B-2019.pdf (2019).

[23] Alberta Energy Regulator (AER), Directive 084, https://aer.ca/documents/directives/Directive084.pdf. (2018).

[24] MacKay K (2019). Fugitive and vented methane emissions surveying on the Weyburn CO_2_-EOR field in southeastern Saskatchewan, Canada. Int. J. Greenh. Gas Control.

[25] Zavala-Araiza D (2017). Super-emitters in natural gas infrastructure are caused by abnormal process conditions. Nat. Commun..

[26] Brandt AR (2014). Methane leaks from North American natural gas systems. Science.

[27] Johnson MR, Tyner DR (2020). A case study in competing methane regulations: Will Canada’s and Alberta’s contrasting regulations achieve equivalent reductions?. Elem. Sci. Anth..

[28] Tyner DR, Johnson MR (2018). A techno-economic analysis of methane mitigation potential from reported venting at oil production sites in Alberta. Environ. Sci. Technol..

[29] Alvarez RA, Pacala SW, Winebrake JJ, Chameides WL, Hamburg SP (2012). Greater focus needed on methane leakage from natural gas infrastructure. Proc. Natl. Acad. Sci. USA.

[30] Masnadi, M. S. *et al.* Global carbon intensity of crude oil production. *Science* **361** (2018). 10.1126/science.aar6859 30166477

[31] Day, S., Dell’Amico, M., Fry, R., Javanmard Tousi, H. Field measurements of fugitive emissions from equipment and well casings in Australian coal seam gas production facilities, (Report to the Department of the Environment, CSIRO), https://publications.industry.gov.au/publications/climate-change/system/files/resources/57e/csg-fugitive-emissions-2014.pdf (2014).

[32] Feitz A (2018). The Ginninderra CH_4_ and CO_2_ release experiment: an evaluation of gas detection and quantification techniques. Int. J. Greenh. Gas Control.

[33] Petrinex. Alberta Public Data https://www.petrinex.ca/PD/Pages/APD.aspx (2018).

[34] Canada Energy Regulator. Energy conversion tables, https://apps.cer-rec.gc.ca/Conversion/conversion-tables.aspx?GoCTemplateCulture=en-CA. (2016)

[35] Clearstone Engineering Ltd. 2018 Alberta upstream oil & gas methane emissions inventory and methodology, https://www.aer.ca/documents/ab-uog-emissions-inventory-methodology.pdf (2019).

