# Supplementary material for: Methane emissions from upstream oil and gas production in Canada are underestimated
Source: Sci Rep. 2021 Apr 13;11:8041. doi: 10.1038/s41598-021-87610-3 (PMC8044210; doi:10.1038/s41598-021-87610-3)
Supplement: Supplementary file 1 — Supplementary Information. [file 41598_2021_87610_MOESM1_ESM.pdf]

Supplemental Materials for: Methane emissions from upstream oil and gas production in Canada are underestimated

Katlyn MacKay[1,2*], Martin Lavoie[1], Evelise Bourlon[1], Emmaline Atherton[1], Elizabeth O'Connell[1], Jennifer Baillie[1], Chelsea Fougère[1], David Risk[1]

[1] Department of Earth Sciences, St. Francis Xavier University, Antigonish, Nova Scotia

[2] Department of Engineering and Applied Science, Memorial University of Newfoundland, St. John's, Newfoundland

*Correspondence to: kmackay@stfx.ca

This pdf file includes:
Figure S1-S2
Table S1-S5

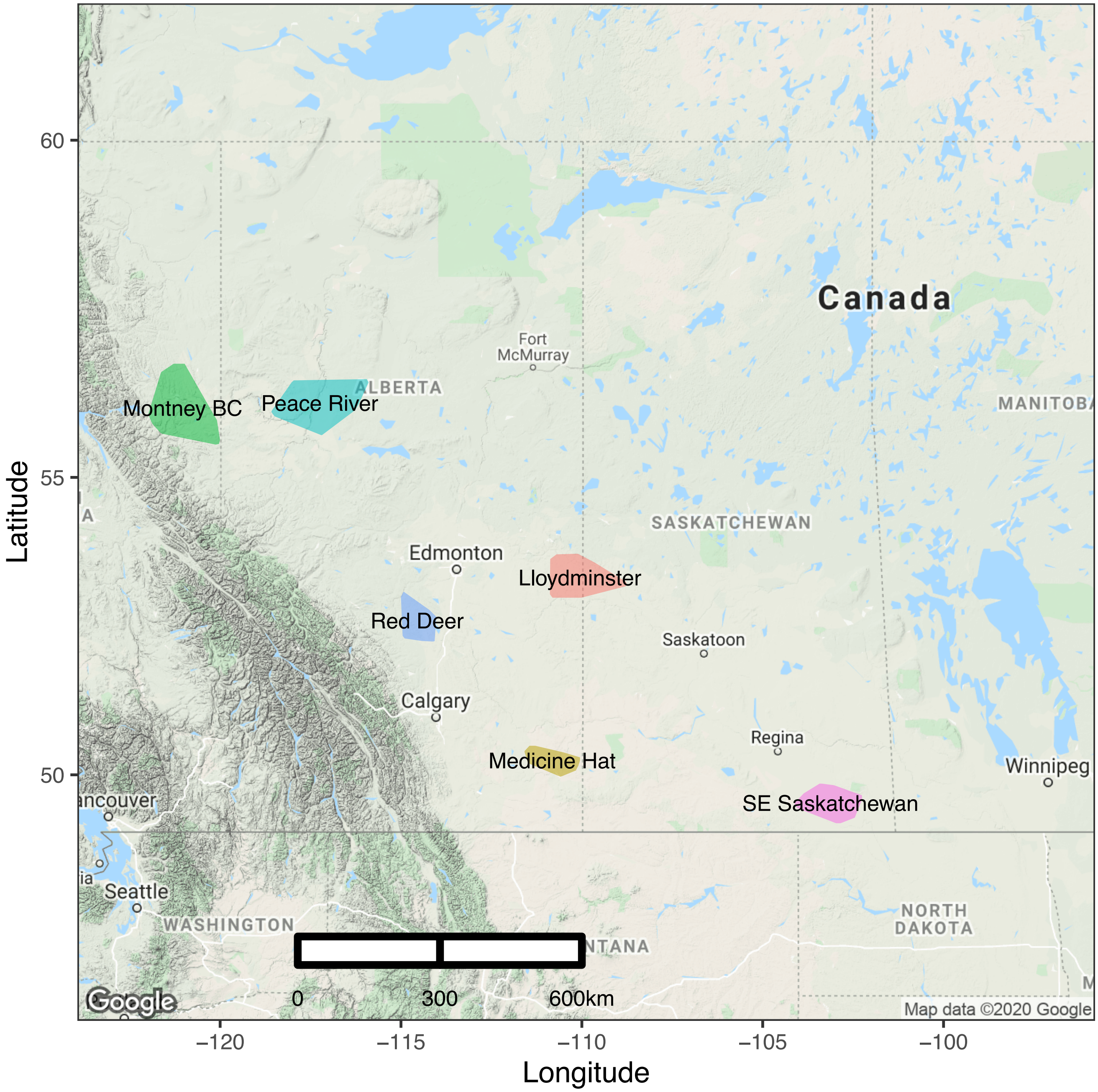

Fig. S1. Polygons showing the geographic regions in British Columbia, Alberta, and Saskatchewan covered in this study. All sites sampled fall within these polygons. Lloydminster and Peace River regions were visited more than once (in different years) (map created using R software version 4.0.0, https://www.r-project.org/).

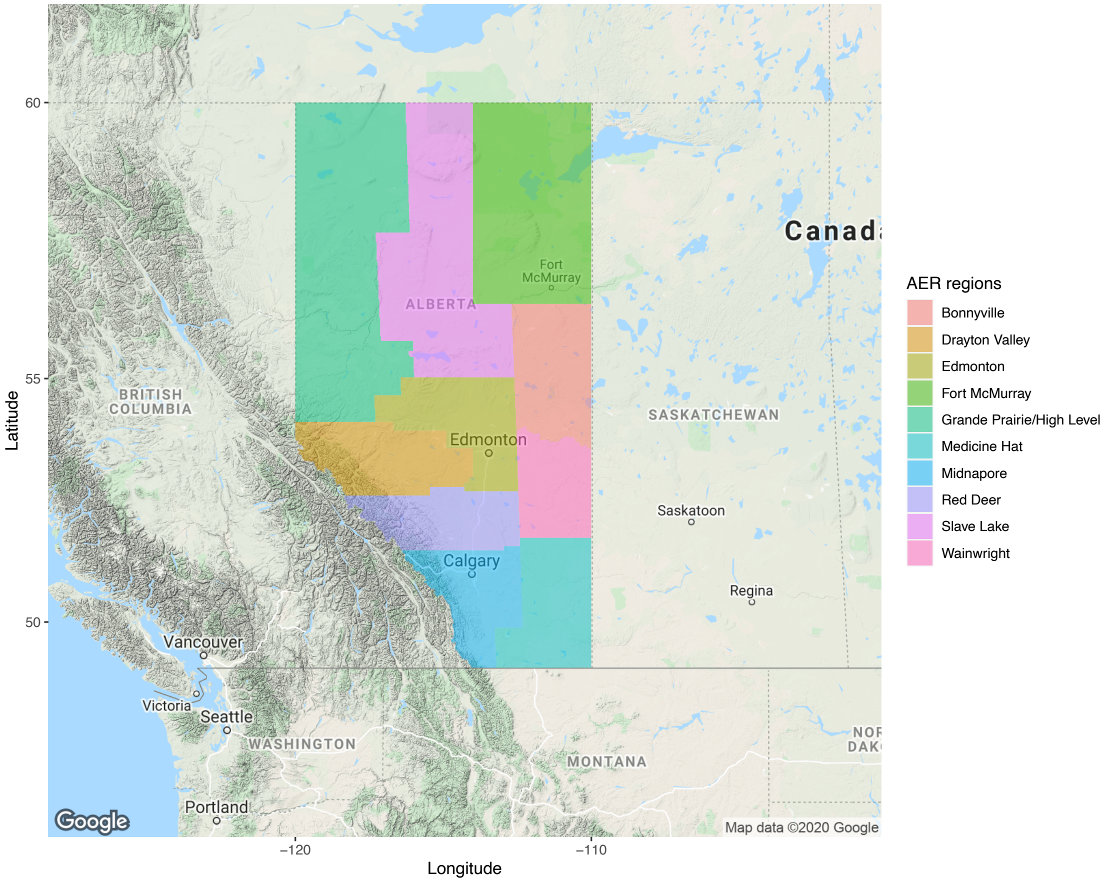

Fig. S2. AER administrative regions for the province of Alberta. These regions (excluding the oilsands dominant region Fort McMurray) are the geographic boundaries used in emission factor calculations and the provincial inventory estimate (map created in R software version 4.0.0, https://www.r-project.org/, using publicly available shapefile from http://www1.aer.ca/ProductCatalogue/649.html).

Region/Campaign	Year	Month(s)	Total surveys	Approx. km surveyed	Publication
SE Saskatchewan	2015	October-November	28	4500	https://dx.doi.org/10.1088/2515-7620/ab01f2
Montney BC	2016	February-March	20	5000	-
Lloydminster (AB side)	2016	October-November	15	2684	https://doi.org/10.1525/elementa.341
Medicine Hat	2016	October-November	15	2881	https://doi.org/10.1525/elementa.341
Peace River	2016	October-November	15	2784	https://doi.org/10.1525/elementa.341
Lloydminster (AB side)	2017	October	15	2600	-
Red Deer	2017	September	15	2600	-
Lloydminster (AB & SK sides)	2018	July	9	2400	-
Peace River	2018	July	3	440	-
All			165	25889	

Table S1. General information for all survey campaigns included in this study. Each survey consisted of multi-hour vehicle-based data collection, with gases being measured every second while driving. All campaigns were conducted on public roads.

Region	Main hydrocarbon produced	Primary Production style(s)	Approx. number of active wells (2020)
SE Saskatchewan	Oil (sour)	Conventional drilling, hydraulic fracturing, EOR	11698
Montney BC	Gas (sweet)	Horizontal drilling, multi-stage hydraulic fracturing	5382
Lloydminster	Heavy oil (sweet)	Cold Heavy Oil Production with Sand (CHOPS)	10571
Medicine Hat	Conventional gas (sweet), light oil	Conventional drilling	20508
Peace River	Heavy oil/bitumen (sour)	Cold Heavy Oil Production (CHOP), thermal recovery	5874
Red Deer	Natural gas/light oil	Conventional drilling, hydraulic fracturing	5648

Table S2. General production information for regions included in this study. Active well counts are based on IHS databases and Fig. S1 polygons are used as geographic boundaries for each region.

**Region**	**MJ/MJ**	**gCO_2e/MJ**
Montney BC	0.0019 ± 0.0013	0.869 ± 0.589
SE Saskatchewan	0.0135 ± 0.0091	6.115 ± 4.145
Lloydminster	0.0706 ± 0.0479	32.084 ± 21.746
Medicine Hat	0.0243 ± 0.0165	11.050 ± 7.489
Peace River	0.0004 ± 0.0003	0.168 ± 0.114
Red Deer	0.0124 ± 0.0084	5.645 ± 3.826

Table S3. Emission intensities in MJ/MJ and gCO_2e/MJ by region. A GWP = 25 and density = 0.678 kg/m^3 (15°C, 1 atm) for CH_4 was used for the gCO_2e calculation.

Campaign/Region	Year	Avg. CH_4 emission rate (m^3/day/site)	Cumulative CH_4 emission rate (m^3/day/region)	Avg. oil production (m^3/day/region)	Avg. gas production (10^3m^3/day/region)
SE Saskatchewan	2015	6.94	7025.73	168.09	348.58
Montney BC	2016	18.01	3835.27	0.00	2006.74
Lloydminster	2016	74.89	83731.58	866.84	139.40
Medicine Hat	2016	13.56	21434.65	312.22	559.16
Peace River	2016	31.47	5286.66	843.52	272.09
Lloydminster	2017	71.62	80076.48	992.53	175.86
Red Deer	2017	29.14	19060.41	279.33	1245.96
Lloydminster	2018	168.45	120778.38	1378.32	174.45
Peace River	2018	11.13	768.00	1478.26	452.00

Table S4. Average site-level CH_4 emission rates, cumulative CH_4 emission rates and average production volumes for sites sampled during each campaign. Production averages correspond to the combined monthly average in which the campaign took place.

AER Region	Site type	EF (m^3 day^{-1})	Regional value†	Number of sites in Alberta[1]
Bonnyville	Bitumen multiple wells	120.3	No	1332
Bonnyville	Bitumen multiwell battery	65.8	Yes	38
Bonnyville	Bitumen single well battery	67.6	Yes	21
Bonnyville	Commingled	11.6	Yes	10
Bonnyville	Compressor station	142.1	No	12
Bonnyville	Gas multiple wells	28.0	No	172
Bonnyville	Gas multiwell battery	43.2	No	521
Bonnyville	Gas single well battery	30.2	Yes	531
Bonnyville	Injection plant	23.2	No	0
Bonnyville	Meter station	10.5	No	160
Bonnyville	Multi battery	178.3	Yes	8
Bonnyville	Oil multiple wells	66.4	No	4
Bonnyville	Oil multiwell battery	116.6	No	0
Bonnyville	Oil single well battery	98.2	No	0
Bonnyville	Regulator station	19.3	No	0
Bonnyville	Single battery	4.0	Yes	274
Bonnyville	Single bitumen well	113.9	No	352
Bonnyville	Single gas well	12.3	No	1561
Bonnyville	Single oil well	38.4	No	0
Bonnyville	Unknown	36.4	No	433
Drayton Valley	Bitumen multiple wells	120.3	No	0
Drayton Valley	Bitumen multiwell battery	142.5	No	0
Drayton Valley	Bitumen single well battery	339.5	No	0
Drayton Valley	Commingled	1.0	Yes	78
Drayton Valley	Compressor station	142.1	No	38

Drayton Valley	Gas multiple wells	28.0	No	1172
Drayton Valley	Gas multiwell battery	43.2	No	215
Drayton Valley	Gas single well battery	138.3	No	882
Drayton Valley	Injection plant	23.2	No	0
Drayton Valley	Meter station	10.9	No	103
Drayton Valley	Multi battery	47.0	No	14
Drayton Valley	Oil multiple wells	66.4	No	736
Drayton Valley	Oil multiwell battery	116.6	No	128
Drayton Valley	Oil single well battery	29.0	Yes	602
Drayton Valley	Regulator station	19.3	No	0
Drayton Valley	Single battery	11.0	Yes	391
Drayton Valley	Single bitumen well	113.9	No	0
Drayton Valley	Single gas well	3.9	Yes	5099
Drayton Valley	Single oil well	9.8	Yes	2701
Drayton Valley	Unknown	24.6	Yes	896
Edmonton	Bitumen multiple wells	120.3	No	0
Edmonton	Bitumen multiwell battery	142.5	No	0
Edmonton	Bitumen single well battery	339.5	No	0
Edmonton	Commingled	69.1	No	17
Edmonton	Compressor station	142.1	No	21
Edmonton	Gas multiple wells	28.0	No	202
Edmonton	Gas multiwell battery	43.2	No	74
Edmonton	Gas single well battery	138.3	No	288
Edmonton	Injection plant	23.2	No	0
Edmonton	Meter station	10.9	No	183
Edmonton	Multi battery	47.0	No	11
Edmonton	Oil multiple wells	66.4	No	450

Edmonton	Oil multiwell battery	116.6	No	104
Edmonton	Oil single well battery	98.2	No	330
Edmonton	Regulator station	19.3	No	0
Edmonton	Single battery	55.9	No	278
Edmonton	Single bitumen well	113.9	No	0
Edmonton	Single gas well	9.9	Yes	1836
Edmonton	Single oil well	38.4	No	1748
Edmonton	Unknown	38.7	Yes	735
Grande Prairie/High Level	Bitumen multiple wells	120.3	No	0
Grande Prairie/High Level	Bitumen multiwell battery	142.5	No	1
Grande Prairie/High Level	Bitumen single well battery	339.5	No	1
Grande Prairie/High Level	Commingled	69.1	No	47
Grande Prairie/High Level	Compressor station	142.1	No	51
Grande Prairie/High Level	Gas multiple wells	28.0	No	1130
Grande Prairie/High Level	Gas multiwell battery	43.2	No	143
Grande Prairie/High Level	Gas single well battery	138.3	No	711
Grande Prairie/High Level	Injection plant	23.2	No	0
Grande Prairie/High Level	Meter station	10.9	No	228
Grande Prairie/High Level	Multi battery	47.0	No	18

Grande Prairie/High Level	Oil multiple wells	66.4	No	668
Grande Prairie/High Level	Oil multiwell battery	116.5	No	50
Grande Prairie/High Level	Oil single well battery	11.6	Yes	162
Grande Prairie/High Level	Regulator station	11.2	Yes	0
Grande Prairie/High Level	Single battery	8.2	Yes	368
Grande Prairie/High Level	Single bitumen well	113.9	No	0
Grande Prairie/High Level	Single gas well	11.0	Yes	5559
Grande Prairie/High Level	Single oil well	38.4	No	2361
Grande Prairie/High Level	Unknown	124.5	Yes	1380
Medicine Hat	Bitumen multiple wells	120.3	No	0
Medicine Hat	Bitumen multiwell battery	142.5	No	0
Medicine Hat	Bitumen single well battery	339.5	No	0
Medicine Hat	Commingled	69.1	No	129
Medicine Hat	Compressor station	57.1	Yes	52
Medicine Hat	Gas multiple wells	32.5	Yes	4773
Medicine Hat	Gas multiwell battery	43.2	No	26
Medicine Hat	Gas single well battery	89.6	Yes	252
Medicine Hat	Injection plant	23.2	No	0
Medicine Hat	Meter station	9.0	Yes	212
Medicine Hat	Multi battery	11.2	Yes	23

Medicine Hat	Oil multiple wells	66.4	No	600
Medicine Hat	Oil multiwell battery	116.5	No	65
Medicine Hat	Oil single well battery	16.0	Yes	462
Medicine Hat	Regulator station	19.3	No	0
Medicine Hat	Single battery	5.4	Yes	347
Medicine Hat	Single bitumen well	113.9	No	0
Medicine Hat	Single gas well	9.5	Yes	35905
Medicine Hat	Single oil well	6.5	Yes	3122
Medicine Hat	Unknown	51.4	Yes	760
Midnapore	Bitumen multiple wells	120.3	No	0
Midnapore	Bitumen multiwell battery	142.5	No	0
Midnapore	Bitumen single well battery	339.5	No	0
Midnapore	Commingled	69.1	No	59
Midnapore	Compressor station	142.1	No	52
Midnapore	Gas multiple wells	28.0	No	1031
Midnapore	Gas multiwell battery	43.2	No	23
Midnapore	Gas single well battery	138.3	No	290
Midnapore	Injection plant	23.2	No	0
Midnapore	Meter station	10.9	No	103
Midnapore	Multi battery	47.0	No	8
Midnapore	Oil multiple wells	66.4	No	158
Midnapore	Oil multiwell battery	116.6	No	40
Midnapore	Oil single well battery	98.2	No	448
Midnapore	Regulator station	19.3	No	0
Midnapore	Single battery	55.9	No	187
Midnapore	Single bitumen well	113.9	No	0
Midnapore	Single gas well	12.3	No	6784

Midnapore	Single oil well	38.4	No	642
Midnapore	Unknown	36.4	No	601
Red Deer	Bitumen multiple wells	120.3	No	0
Red Deer	Bitumen multiwell battery	142.5	No	0
Red Deer	Bitumen single well battery	339.5	No	0
Red Deer	Commingled	5.3	Yes	102
Red Deer	Compressor station	142.1	No	83
Red Deer	Gas multiple wells	6.8	Yes	435
Red Deer	Gas multiwell battery	56.1	Yes	270
Red Deer	Gas single well battery	122.7	Yes	1264
Red Deer	Injection plant	23.2	No	0
Red Deer	Meter station	2.9	Yes	155
Red Deer	Multi battery	47.0	No	26
Red Deer	Oil multiple wells	66.4	No	449
Red Deer	Oil multiwell battery	28.5	Yes	139
Red Deer	Oil single well battery	12.0	Yes	750
Red Deer	Regulator station	19.3	No	0
Red Deer	Single battery	39.7	Yes	505
Red Deer	Single bitumen well	113.9	No	0
Red Deer	Single gas well	4.5	Yes	4758
Red Deer	Single oil well	22.6	Yes	2334
Red Deer	Unknown	21.8	Yes	1127
Slave Lake	Bitumen multiple wells	8.7	No	482
Slave Lake	Bitumen multiwell battery	21.4	Yes	193
Slave Lake	Bitumen single well battery	7.8	Yes	57
Slave Lake	Commingled	69.1	No	0
Slave Lake	Compressor station	142.1	No	8

Slave Lake	Gas multiple wells	28.0	No	56
Slave Lake	Gas multiwell battery	43.2	No	0
Slave Lake	Gas single well battery	138.3	No	12
Slave Lake	Injection plant	23.2	No	0
Slave Lake	Meter station	10.9	No	62
Slave Lake	Multi battery	47.0	No	4
Slave Lake	Oil multiple wells	66.4	No	105
Slave Lake	Oil multiwell battery	116.6	No	36
Slave Lake	Oil single well battery	30.6	Yes	271
Slave Lake	Regulator station	19.3	No	0
Slave Lake	Single battery	2.9	Yes	146
Slave Lake	Single bitumen well	113.9	No	165
Slave Lake	Single gas well	12.3	No	389
Slave Lake	Single oil well	38.4	No	959
Slave Lake	Unknown	2.4	Yes	318
Wainwright	Bitumen multiple wells	120.3	No	24
Wainwright	Bitumen multiwell battery	352.8	Yes	153
Wainwright	Bitumen single well battery	502.4	Yes	425
Wainwright	Commingled	456.0	Yes	38
Wainwright	Compressor station	142.1	No	19
Wainwright	Gas multiple wells	28.0	No	112
Wainwright	Gas multiwell battery	43.2	No	8
Wainwright	Gas single well battery	308.2	Yes	180
Wainwright	Injection plant	35.2	Yes	128
Wainwright	Meter station	10.9	No	125
Wainwright	Multi battery	40.3	Yes	9
Wainwright	Oil multiple wells	75.0	Yes	1046

Wainwright	Oil multiwell battery	135.8	Yes	257
Wainwright	Oil single well battery	115.2	Yes	1154
Wainwright	Regulator station	25.2	Yes	0
Wainwright	Single battery	65.3	Yes	417
Wainwright	Single bitumen well	190.0	Yes	73
Wainwright	Single gas well	357.2	Yes	3795
Wainwright	Single oil well	74.8	Yes	3227
Wainwright	Unknown	26.3	Yes	611
Total				122254

[1]Active site counts are based on IHS infrastructure databases and 2018 Petrinex volumetric reporting data

[†]A "Yes" in this column means that the EF was calculated using only measurements from the specified sites within the specified region. "No" means that the EF was calculated using an average of measurements for that specific site type across all AER regions (i.e. the entire Alberta dataset).

Table S5: Site-level emission factors (EFs) for all AER regions in Alberta used to derive the total inventory estimate. Where possible, EFs were calculated using site- and region-specific measurements.
